# Supplementary figures and images for: Manipulation of the nuclear envelope-associated protein SLAP during mammalian brain development affects cortical lamination and exploratory behavior
Source: Biol Open. 2024 Mar 11;13(3):bio060359. doi: 10.1242/bio.060359 (PMC10958201; doi:10.1242/bio.060359)

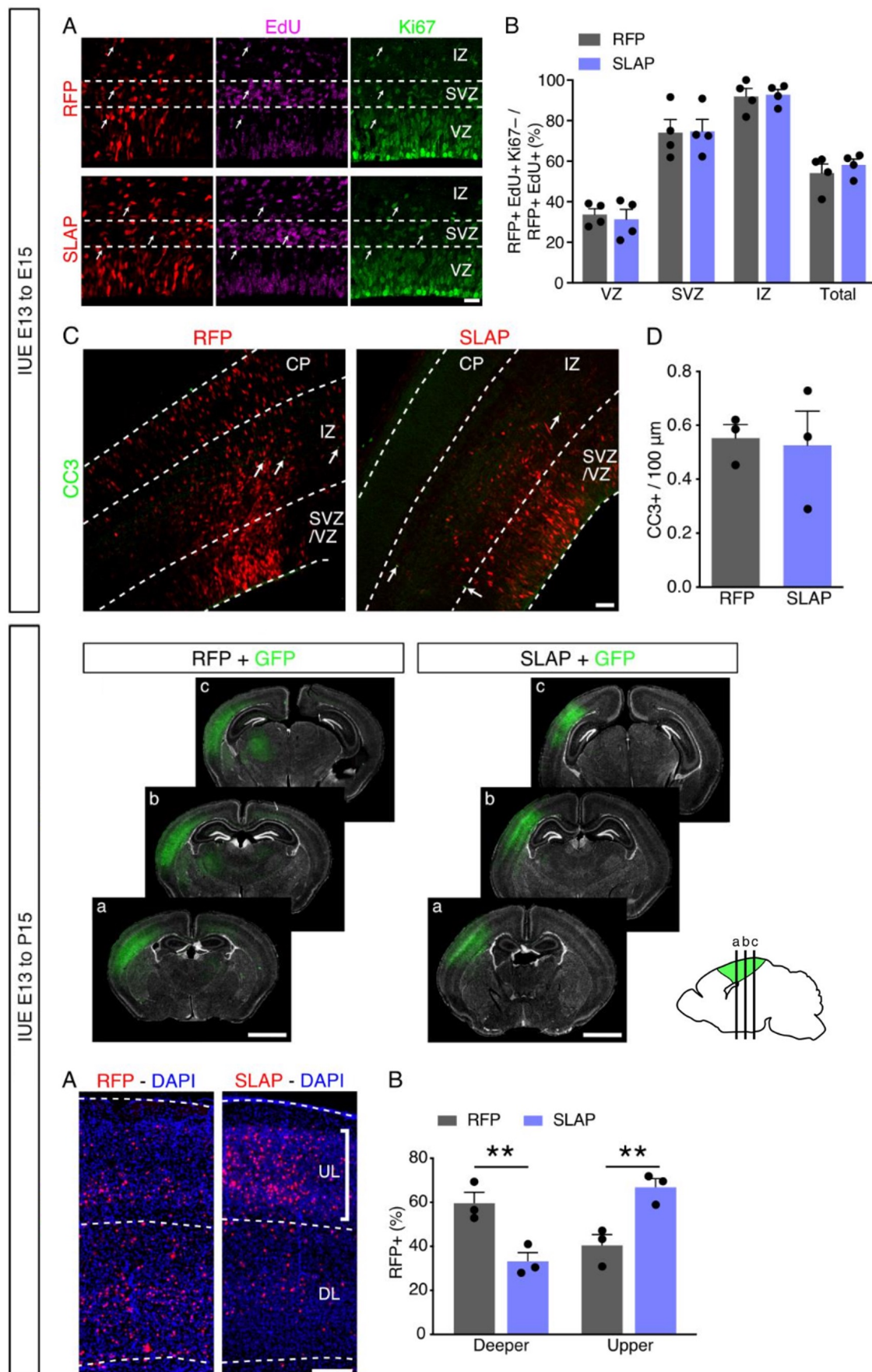

Fig. S1.

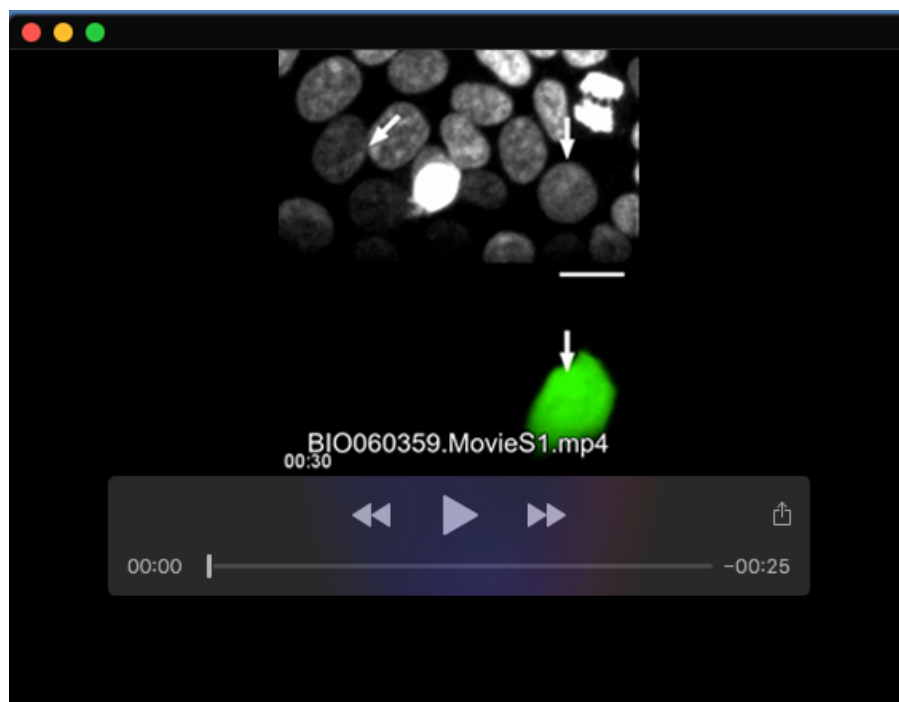

**Movie 1.**

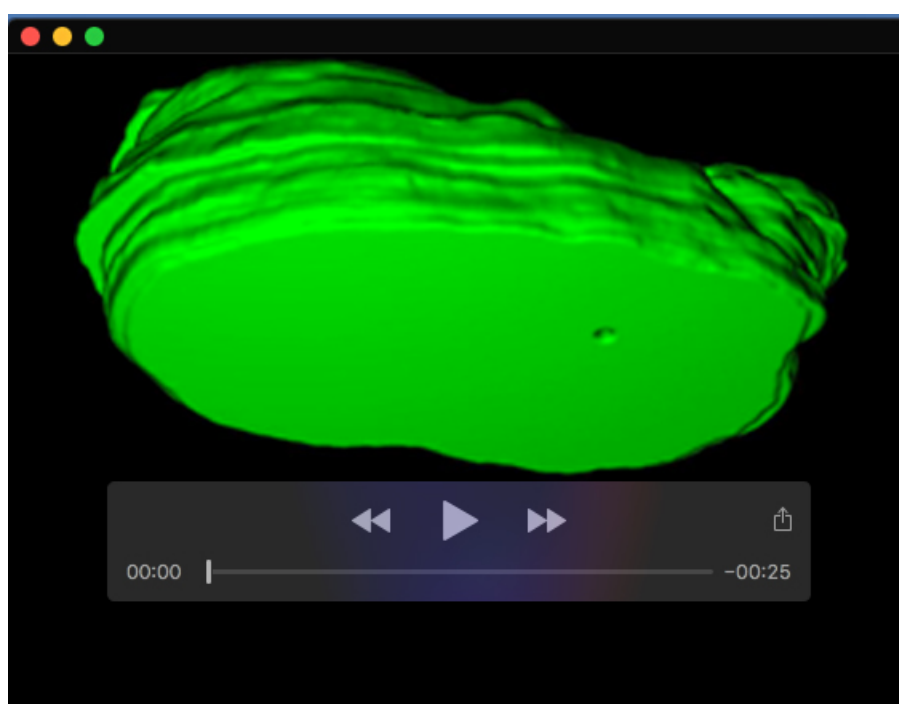

**Movie 2.**

Supplement: Supplementary information [file biolopen-13-060359-s1.pdf]
